# Association of Public Awareness and Knowledge of Climatic Change With Sociodemographic Factors

**DOI:** 10.7759/cureus.47381

**Published:** 2023-10-20

**Authors:** Prashanth K Vishwakarma, Sanjay Vaghmare, Satyabrat Banerjee, Aruna P Vishwakarma, Alka Waghmare, Anoli Agrawal, Manish Sharma

**Affiliations:** 1 Department of Public Health Dentistry, Jawahar Medical Foundation's Annasaheb Chudaman Patil Memorial (JMF's ACPM) Dental College, Dhule, IND; 2 Department of Ophthalmology, Jawahar Medical Foundation's Annasaheb Chudaman Patil Memorial (JMF's ACPM) Dental College, Dhule, IND; 3 Department of Conservative Dentistry and Endodontics, Jawahar Medical Foundation's Annasaheb Chudaman Patil Memorial (JMF's ACPM) Dental College, Dhule, IND; 4 Department of Pediatric Dentistry, Jawahar Medical Foundation's Annasaheb Chudaman Patil Memorial (JMF's ACPM) Dental College, Dhule, IND; 5 Department of Periodontics, Jawahar Medical Foundation's Annasaheb Chudaman Patil Memorial (JMF's ACPM) Dental College, Dhule, IND; 6 Department of Oral Pathology, Jawahar Medical Foundation's Annasaheb Chudaman Patil Memorial (JMF's ACPM) Dental College, Dhule, IND

**Keywords:** perception, urban, awareness, knowledge, climatic change

## Abstract

Introduction

The earth has experienced significant shifts in climate patterns over the past few years. The main aim of this investigation was to establish the association between the sociodemographic factors on the extent of knowledge, perspectives, and awareness of the urban population in Delhi and the phenomenon of climate change.

Materials and methods

This study was conducted on 1,200 individuals residing in Delhi, India, who were given a well-organized validated questionnaire to gather data. The relationship between different factors influencing awareness and climate change was evaluated using the chi-square test.

Results

The investigation's findings revealed that the younger generations exhibited heightened consciousness as a result of the impact of education and social media, both of which possess an exceedingly significant role in the dissemination of awareness. Additional elements that influenced the participants' awareness regarding climate change encompassed their educational attainment, profession, and financial resources, which were noticeably more advantageous for the upper and upper-middle social strata. A majority of the respondents, amounting to 65%, hailed from the middle class, with 61% of them holding degrees. The majority of the respondents were well-informed about climate change, with a predominant percentage falling within the age range of 21-40 years (77%) and over the age of 61 years (73%). Notably, 92% of the respondents belonging to the upper class exhibited awareness of climate change. About 52% of the respondents expressed a moderate level of concern towards climate change.

Conclusions

The analysis revealed that most individuals possessed knowledge regarding the impact of climate change on their way of life. Consequently, they acknowledged the significance of acquiring a more comprehensive understanding of climate change.

## Introduction

Climate change is a global predicament, primarily attributable to human activities, bolstered by well-established evidence-based research conducted by relevant experts [1]. The escalating greenhouse gas effect induces alterations in temperature, glacier melting, and sea level elevation. Its repercussions permeate all domains of existence, encompassing human well-being, provisions of sustenance, water provision, agriculture, and energy sources. Thus, it is imperative to critically confront these climate change-related quandaries. This approach entails the identification of climate change impacts, formulation of policies, and subsequent implementation of technological modifications.

The urban agglomeration of Delhi ranks second in size, following Mumbai, among the cities in India. It is undeniable that Delhi, being a city, is susceptible to the potential impacts of climate change. The alterations in Delhi's climate are primarily attributed to the persistent and unceasing discharge of immense quantities of greenhouse gases (CO_2_, CH_4_) into the atmosphere. This emission is predominantly a consequence of human activities, including the combustion of fossil fuels, the existence of landfills, and the presence of heavy traffic. These activities are thought to be modifying the climate system of Delhi.

Global warming has resulted in a substantial rise in temperature over recent years, leading to an escalation in natural disasters such as hurricanes, floods, storms, lightning strikes, heat strokes, and rainstorms [2]. This imposition is a significant burden on both the economy and society. Climate plays a role in its devastation due to the global sequence of events. In recent years, as a consequence of drastic alterations in weather patterns, individuals have become more cognizant of climate change, instigating numerous endeavors that advocate for societal change. The primary cause of the worldwide increase in temperature is the burning of fossil fuels, which account for up to 80% of all global industrial energy consumption [3]. Climatic alterations have an impact on physical surroundings, health systems of individuals, and human civilization. Changes in the climate system have led to temperature fluctuations that influence human settlements, societies, natural environments, and wildlife.

The concept of a "climate crisis" can encompass both the projected and actual adverse consequences of climate change. While adaptation primarily depends on the availability of climate change-related information, voluntary mitigation is driven by the anticipation of susceptibility to hazards and the gravity of climate change or climatic variability impacts [4]. Fundamental knowledge significantly alleviates concerns about climate change and fosters the implementation of environmentally conscious policies. It is of utmost importance to cultivate awareness among individuals who have yet to encounter the concept of climate change. Furthermore, future generations should be educated about climate change and its profound implications in educational settings [5]. The national and local initiatives designed to enhance citizens' dedication to climate change should be tailored to the unique circumstances of each nation, particularly the developing world. Despite recent efforts to foster a comprehensive public understanding of climate change, this issue remains inadequately understood. It is imperative to identify the factors that influence an individual’s awareness. The objective of this study was to evaluate the degree of climate change awareness within the urban population of Delhi and its correlation with various sociodemographic factors that impact respondents' awareness. The null hypothesis posited that there would be no significant association between sociodemographic factors influencing awareness and climate change awareness among respondents.

## Materials and methods

Study method and design

A qualitative research methodology was selected to examine and gather essential data. The study was structured as a cross-sectional observational study in the Department of Public Health Dentistry, in which data were acquired from the study participants from March to July 2022. The cross-sectional study design was chosen to examine a large population at a given point in time. The study was approved by the Institutional Ethical Committee (EC/NEW/INST/2022/2959/231) and was conducted in accordance with the Declaration of Helsinki. Written informed consent was obtained from all participants, after explaining the purpose of the survey to the participants. The act of participating was completely voluntary, and no monetary incentives or other forms of compensation were provided to motivate participants to take part. The survey was carried out in an anonymous manner in order to account for the Hawthorne effect and information bias.

Study area

The specific demographic under investigation is the Delhi region. According to the 2011 census, the population of Delhi was 11,007,835 individuals. The study area is situated at a latitude of 28.644800 and a longitude of 77.216721 in Delhi, India. This investigation was conducted across different sectors within the vicinity of the municipal government.

Study unit

Data were gathered from individuals who served as heads of household, with an age threshold of ≥18 years. This information was obtained through three series of planned interviews wherein participants were asked about their understanding and perspectives regarding climate change. The data for this research study were obtained by means of face-to-face interviews conducted with the participants by the research team of six members from the Public Health Department who were trained and tested with a pilot study to ask uniform questions from the participants.

Sample size estimation

The necessary sample size was determined using the following equation, where n represents the required sample size, the confidence interval was set at 95%, Z was equal to 1.96, and E denotes the margin of error at 5%. The formula used for analysis was (Z-score)^2^ × standard deviation (SD) x (1-SD)/(margin of error)^2^. The population proportion was maintained at 50% because it is the fraction that provides the maximal sample size [5]. Consequently, 1,068 participants were deemed necessary for the study. To conduct the questionnaire survey, 1,200 individuals hailing from the Delhi capital region were included. The selection of respondents for the study was accomplished using simple random sampling during the household survey.

Inclusion criteria

Participants must be at least 18 years of age and have resided in a residential dwelling in Delhi, India for a minimum of one year. These dwellings should possess fundamental and indispensable amenities. Additionally, individuals who willingly provided their consent to partake in the research were included as subjects.

Exclusion criteria

The exclusion criteria for this study included individuals who were below the age of 18, above the age of 75, and those who were unable to communicate or understand the questionnaire across all age groups. The maximum age limit was chosen as 75 years, which is the average life expectancy of an individual. 

Tools and technique

The questionnaire was formed in consultation with five experts from public health with more than 10 years of experience, who were not involved in the study. A meticulously crafted survey was developed to accomplish the research objectives, drawing on content validity. A preliminary examination or pretesting of the questionnaire was carried out on 140 individuals who were not involved in the study to evaluate the dependability of the inquiries. The reliability of the questionnaire was tested using Cronbach’s alpha, which was 0.86. The questionnaire was retested after a period of one month using the same cohort to assess the level of agreement among the questions. The inter-observer agreement was assessed using the kappa coefficient, which was 0.92.

The survey served as the primary instrument for data collection. It was divided into two distinct sections. The first section, known as Part A, focused on sociodemographic characteristics such as age group, gender, education level, occupation, combined household income, and marital status. The second section, Part B, comprised closed-ended questions pertaining to knowledge and awareness about climate change. The bilingual questionnaire was scrutinized by language experts, who translated the questions into Hindi. Subsequently, these translations were back-translated into English by two independent language experts. 

Statistical analysis

Data obtained from the questionnaire were entered into an Excel spreadsheet to serve as a database. The collected data were statistically analyzed using IBM SPSS Statistics for Windows, Version 22 (Released 2013; IBM Corp., Armonk, New York, United States). The Shapiro-Wilk test was used to check the normality of the data. Frequency distributions and tables were employed to summarize and present both sociodemographic variables and participants' responses. Cross-tabulation was used to examine potential associations between the variables. To determine the significance between variables, either chi-square or Fisher's exact tests were employed, depending on whether the expected cell frequencies were less than or equal to five. Additionally, the strength of the association was assessed using the Phi test.

## Results

Our investigation rejected the null hypothesis, and meaningful connections between the diverse factors influencing awareness and climate change were revealed. The study involved a total of 1,200 participants, of whom 1,094 individuals completed the questionnaires in their entirety. Partially completed questionnaires that were not fully answered by the participants were deemed ineligible for inclusion in the research analysis.

Demographic characteristics of the respondents

In accordance with fundamental data regarding the characteristics of the respondents who participated in our study, it was observed that 59% of the respondents fell within the age range of 21 to 40 years. Furthermore, 56% of respondents were males, and 44% of respondents were females in the study population. The individuals who participated in the study were predominantly from middle-class socioeconomic backgrounds (65%). Moreover, a considerable majority of the respondents possessed a degree or higher level of educational attainment, amounting to 61% of the total sample. A substantial proportion of the respondents identified themselves as students, comprising 32% of the total sample size, while 30% indicated that they were private employees. In terms of annual income, the majority of respondents earned between 1 and 3 lakhs per year, accounting for 42% of the total sample population. This information is presented in Table 1 as a comprehensive summary of the respondents’ demographic profiles.

**Table 1:** Frequency and percentage distribution of the sociodemographic variables of the respondents

Sociodemographic variables (n = 1,094)	Frequency	Percentage from total population	Male	Male %	Female	Female %
Age (in years)						
Less than 21	184	17	141	77	43	23
21-40	642	59	364	57	278	43
41-60	203	19	131	65	72	35
More than 61	65	6	54	83	11	17
Gender	1,094	--	612	56	482	44
Socioeconomic class						
Upper class	327	30	210	64	117	36
Upper middle class	532	49	322	61	210	39
Lower middle class	177	16	121	68	56	32
Lower class	58	5	34	59	24	41
Highest education qualification						
Illiterate	8	1	3	38	5	63
Below 10th standard	18	2	11	61	7	39
10th standard	83	8	50	60	33	40
12th standard	210	19	134	64	76	36
Diploma	112	10	67	60	45	40
Degree	663	61	397	60	266	40
Occupation						
Employed for daily wages	86	8	65	76	21	24
Government employee	87	8	55	63	32	37
Private employee	329	30	220	67	109	33
Self-employed/business	82	7	72	88	10	12
Homemaker	94	9	5	5	89	95
Student	352	32	222	63	130	37
Retired	48	4	41	85	7	15
Unemployed	16	1	13	81	3	19
Combined household annual income (in INR)						
Below 50,000	46	4	40	87	6	13
50,000-1 lakh	273	25	191	70	82	30
1-3 lakhs	458	42	272	59	186	41
3-5 lakhs	191	17	102	53	89	47
Above 5 lakhs	126	12	85	67	41	33

Association between sociodemographic characteristics and awareness

Cross-tabulation, a statistical technique used to explore the relationship between different variables, was conducted to investigate the correlation between sociodemographic factors and the level of awareness of climate change. The various sociodemographic variables considered in this analysis, including age, gender, educational background, occupation, and family income, displayed a significant and meaningful association with climate change, as indicated by the statistical findings presented in Table 2.

**Table 2:** Cross-tabulation association between sociodemographic variables and awareness of climate change using chi-square test *p < 0.05: Significant; **p < 0.001: Highly significant; df: Degree of freedom; x2: chi-square value

Sociodemographic variables	Awareness of climate change		Chi-square test
	Yes %	No %	
Age (in years)			
Less than 20	65	15	χ2 = 12.105, df = 6, p = 0.041*
21-40	77	9	
41-60	68	12	
More than 61	73	11	
Gender			
Male	73	14	χ2 = 24.180, df = 2, p < 0.001**
Female	72	8	
Socioeconomic status			
Upper	92	4	χ2 = 56.433, df = 6, p < 0.001**
Upper middle	73	8	
Lower middle	66	17	
Lower	49	32	
Qualification			
Illiterate	42	44	χ2 = 91.613, df = 10, p < 0.001**
Below 10th standard	58	36	
Metric school	61	29	
Secondary school	67	13	
Certification	57	14	
Graduate	79	7	
Employment			
Daily wages	54	17	χ2 = 41.890, df = 14, p < 0.001**
Government job	84	7	
Private job	77	6	
Self-employed/business	68	21	
Homemaker	63	14	
Student	74	11	
Retired	81	8	
Unemployed	59	14	
Total annual income (in INR)			
Below 50,000	80	18	χ2 = 62.364, df = 8, p < 0.001**
50,000-1 lakh	65	16	
1-3 lakhs	68	11	
3-5 lakhs	80	7	
Above 5 lakhs	92	6	

The aforementioned percentages seem to indicate that the younger demographic of age 21-40 years, which accounted for 77% of the respondents, as well as the retired generation, which constituted 73%, displayed a higher level of awareness than the age group of 41-60 years. Similarly, it was observed that individuals who held secure government jobs, comprising 84% of the respondents, as well as those belonging to the upper class (92%), exhibited a greater degree of concern regarding climate change than the remaining participants. Furthermore, an examination of gender associations revealed that both males and females displayed a similar level of awareness, with 73% of males and 72% of females demonstrating knowledge and concern regarding climate change. In terms of educational attainment, an overwhelming majority of respondents (79%) possessed a degree or higher, indicating a significant level of understanding and knowledge pertaining to climate change.

Knowledge and awareness of respondents about climatic change

The association between awareness and the phenomenon of climatic change became more pronounced as there was a significant increase in the annual income of families. It is worth noting that a considerable majority (73%) of the respondents participating in the study possessed an understanding of the term "climate change" and its implications, in contrast to a mere 11% who had never come across this term and another 16% who remained non-responsive when queried about it. Furthermore, 90% of the respondents expressed a keen interest in expanding their knowledge about climate change and the various ramifications it entails. Additionally, 88% of the survey participants indicated that climate change adversely affected their way of life, as detailed in Table 3.

**Table 3:** Percentage of responses of the respondents on climate change awareness

Questions	Yes %	No %
Are you aware of the term “climate change”	73	11
Climate change affecting lifestyle or living habits	88	5
Do you wish to know more about climate change's impact on health risk	90	10

Most respondents, comprising 38% of the total sample, exhibited an overwhelming degree of concern regarding the pressing issue of climate change. This profound sense of concern, which was evinced by a significant majority of respondents, underscores the gravity and magnitude of the matter at hand. Furthermore, 52% of the respondents expressed a level of concern that can be characterized as fairly significant, indicative of a growing awareness and acknowledgment of the imminent dangers and potential consequences associated with climate change. It is worth noting, however, that a small minority of participants, constituting only 9% of the total sample size, exhibited a sense of uncertainty regarding climate change. This fraction of respondents, while comparatively small, underscores the complexity of the issue, as it reveals the presence of varying perspectives and levels of comprehension among the respondents. Moreover, it is important to acknowledge the existence of a negligible percentage (approximately 1%) of respondents who displayed a fair amount of unconcern about the matter. Although this percentage is relatively insignificant in the grand scheme of this study, it serves as a reminder that there still exist individuals who may not fully grasp the gravity and urgency of the climate change crisis. The tabulated data presented in Table 4 further elucidate the distribution and representation of these differing outlooks and attitudes towards climate change among the respondents.

**Table 4:** Percentage of respondents concerned about climatic change

Concern about climate change	Percentage
Strongly concerned	38
Fairly concerned	52
Uncertain	9
Fairly unconcerned	1
Strongly unconcerned	1

## Discussion

Knowledge of the respondents about climate change

This research aims to ascertain the understanding, perspectives, and consciousness of individuals residing in the communities of Delhi regarding the phenomenon of climate change. The discoveries derived from this investigation offer valuable insights into the thoughts and convictions of individuals based on their initial encounters at the local level. Residents of urban areas are affected by climate change. Climate change is a relatively recent and intricate topic, often accompanied by misinformation, necessitating the inevitable creation of awareness in the battle against it [6]. Delhi lies in the landlocked (Uttar Pradesh, Haryana, and Rajasthan) northern plains of the Indian subcontinent, and its weather and climate are greatly influenced by its proximity to the Himalayas and Thar Desert of Rajasthan. Analyses of meteorological data in Delhi have already unveiled noteworthy disparities in patterns pertaining to the minimum and maximum temperatures, alongside cloud quantities and relative humidity. Given the escalating concentrations of greenhouse gases in the atmospheric composition of Delhi, it is postulated that the climate may undergo an increase in temperature ranging from 2°C to 4°C [2,7]. 

Awareness of the term climate change and factors affecting awareness

Our investigation revealed a significant correlation between awareness of climatic variation and variables such as age, sex, occupation, and family income. Our results align with prior studies, which indicated that the majority of respondents had satisfactory comprehension of the subject matter [8,9]. The rationale behind the surge in consciousness among young individuals and retirees in our analysis can potentially be attributed to the influence of social media and printed publications [10]. Recently, as a consequence of the presence of social media platforms and diverse campaigns, individuals have become cognizant of the issue of climate change and are amenable to altering their lifestyle choices to confront this environmental predicament. Additionally, it has been posited that an individual's cultural background serves as a significant predictor of their level of concern regarding climate change, rather than relying solely on information acquired from media sources [9]. Individuals who are employed in positions that provide job security, belong to a higher social class, and have a family income exceeding five lakhs demonstrated a greater level of awareness concerning the subject matter in comparison to others. This heightened awareness can potentially be attributed to their improved financial stability and the accessibility of resources at their disposal.

Climate change and health are two topics that the World Health Organization has tackled through a multitude of approaches. Among these approaches are an extensive compilation of policy summaries, recommendations, resources, and instructional guides; active involvement in diverse initiatives to elevate the significance of health concerns within the climate discourse; and advocacy endeavors and multimedia materials aimed at policymakers as well as the broader public [11]. Various national and regional initiatives tailored to the requirements of each nation should be enforced. To alter conduct and acquire communal backing for the necessary measures aimed at mitigating greenhouse gas emissions, it is imperative to amplify awareness and comprehension regarding the repercussions of climate change on public health, as well as to persuade healthcare professionals to endorse endeavors aimed at reducing vulnerability and enhancing well-being, such as measures to mitigate and adapt [12].

Our investigation revealed that contemporary young individuals possess a high level of awareness of the impact of alterations in climate patterns on their existence. The primary cause of this phenomenon is the modification of our educational guidelines. Recently, due to mounting anxiety surrounding climate change, it has become compulsory within educational institutions to instruct pupils on the alterations transpiring in our surroundings as a result of the greenhouse effect and the ensuing repercussions on our physical and mental conditions [13]. In our study, a considerable proportion of respondents (approximately 52%) exhibited a notable level of concern regarding the potential consequences of climate change in their daily existence and natural surroundings. This aligns with the outcomes reported by Davydov and Mikhailova, who observed that 50% of participants experienced an influence on their daily lives [14].

Despite the relatively high level of literacy in India, specifically in Delhi, which stands at approximately 89% according to the Census India of 2011, a significant majority of around 90% of individuals express a desire to further educate themselves on the subject of climate change and its ramifications [15]. This inclination is accompanied by a prevailing mentality within the population that discourages them from actively engaging in government policies. Moreover, the decisions made by authorities fail to resonate with the everyday struggles of ordinary citizens who are solely focused on meeting their basic needs on a day-to-day basis [16]. According to the World Poll, the level of public cognizance of climate change as a menacing peril is exceedingly elevated (80-89%). Despite individuals possessing knowledge of climate change, their apprehensions regarding this matter are remarkably feeble. It is crucial to inculcate consciousness in the general population.

Recommendations for society

The primary objective should be to establish a strong connection between individuals' knowledge and concerns by means of public education. Enhancing one's awareness is crucial for the improvement of comprehension regarding emerging trends and evolving concepts. Encouraging the adoption of recycling habits and active participation in community activities is imperative. In order to effectively adapt to climate change, disease surveillance at the community level must be implemented as part of the necessary measures. Furthermore, it is essential to provide opportunities for public health officials to educate the general public on the health risks associated with climate change, as well as the actions required to mitigate climate change and successfully adapt to its risks.

Limitations of the study

One constraint of this study is that the male population is slightly overrepresented in the sample, resulting in a gender distribution imbalance. However, it can be posited that the findings remain unaffected by this marginal disparity and that the observed patterns would persist in a sample with equal representation of both genders. Subsequent investigations may explore the level of climate change awareness across various nations. 

## Conclusions

The findings of this study indicate that 73% of survey respondents possessed knowledge of climate change, and 88% of the respondents expressed a strong conviction that alterations in climatic patterns are undeniably exerting a profound impact on their personal existence. About 90% of respondents want to know more about climatic change and its effects. However, 11% of respondents were unfamiliar with climatic change. This study reveals that the urban sector exhibits a commendable literacy rate in terms of school enrolment. Individuals with higher education possess sufficient awareness of climate change, thereby highlighting the importance of education. Moreover, the upper and upper middle classes, benefiting from greater access to resources, exhibited a positive correlation between awareness and climate change. Consequently, a more comprehensive understanding of the dynamic nature of climatic effects has the potential to shape individual perspectives. 
